# A rare case report of orchiopexy and hysterectomy in an Afghan boy with persistent Müllerian duct syndrome

**DOI:** 10.1016/j.ijscr.2024.109235

**Published:** 2024-01-10

**Authors:** Dawood Hossaini, Mohammad Mahdi Wahdat, Ali Aklaqi, Murtaza Haidary

**Affiliations:** a Department of Biology and Microbiology, School of Medical Laboratory Technology, Khatam Al- Nabieen University, Kabul, Afghanistan; b Faculty of Medicine, Khatam -Al Nabieen University, Kabul, Afghanistan; c Medical Research and Technology Center, Khatam Al Nabieen University, Kabul, Afghanistan

**Keywords:** Persistent Müllerian duct syndrome, Afghan boy, Hysterectomy, Orchidopexy, Case report

## Abstract

**Introduction:**

Persistent Müllerian duct syndrome (PMDS), a rare genetic aberration, is characterized by the presence of Müllerian duct (MD) features in males. PMDS is usually caused by a defect in the Müllerian inhibitory system and is discovered during surgical interventions.

**Case report:**

We present the case of a 14-year-old Afghan boy with severe abdominal pain who was initially diagnosed with bilateral undescended testicles and abdominal complex cysts. He was supposed to undergo a cystectomy and orchiopexy surgery. During the surgical intervention, an unexpected finding was made whereby fibrotic-like ovaries, fallopian tubes, and a segment of the uterus were identified, ultimately leading to the diagnosis of PMDS. The MD was carefully removed, and the testicles were delicately repositioned during an orchiopexy procedure.

**Discussion:**

In our case, the patient exhibited bilateral undescended testicles along with fibrotic-like ovaries, fallopian tubes, and a portion of the uterus, representing the presence of the female type of PMDS. To safeguard fertility, orchidopexy is recommended for pediatric patients. Conversely, in the older age group, orchidectomy is advised as a precautionary measure against the heightened susceptibility to testicular carcinoma.

**Conclusion:**

PMDS can be associated with an undescended testicle and abdominal pain. Hence, it is crucial to thoroughly evaluate patients who have undescended testes for the presence of PMDS, and surgeons must maintain a heightened sense of awareness for PMDS while exploring individuals who present with bilateral undescended testes, as exemplified in our case.

## Highlights

• Persistent Müllerian duct syndrome (PMDS) is a rare genetic defect characterized by the presence of Müllerian duct derivatives in phenotypically and genotypically male individuals.
• PMDS poses a potential risk of malignancy in Mullerian structures if left untreated.
• PMDS is typically identified during orchiopexy or inguinal hernia repair.
• Surgeons should exercise a high level of suspicion for PMDS when evaluating individuals with undescended testicles.

## 1 Intoduction

Persistent Müllerian duct syndrome (PMDS) is an uncommon hereditary disorder characterized by the incomplete regression of the Müllerian duct structures, such as fallopian tubes, uterus, and upper vagina, within an individual who otherwise exhibits typical male physical and genetic traits (46, XY) [1]. The inheritance patterns of PMDS include X-linked, autosomal dominant, and autosomal recessive inheritance [2]. Approximately 85 % of affected people carry mutations in either the Mullerian inhibiting substances (MIS) gene on chromosome 19p13 or the MISR II gene on chromosome 12q13. However, in the remaining 15 % of cases, the cause of PMDS remains unknown [3]. Patients often present with a unilateral or bilateral undescended testicle, accompanied by an inguinal hernia [4]. While the manifestations of PMDD can vary, infertility is the most common complication experienced by individuals with this condition [5]. The complexity and anatomical variability of PMDS present significant diagnostic challenges [2]. The prevalence of PMDS is not well established, given its rarity, with fewer than 300 cases documented in the literature [6]. Only one case has been reported from Afghanistan [7]. Herein, we highlight the case of PMDS in an Afghan boy presented with bilateral undescended testicles, aligning with the SCARE criteria [8].

## 2 Case report

**Fig. 1:**
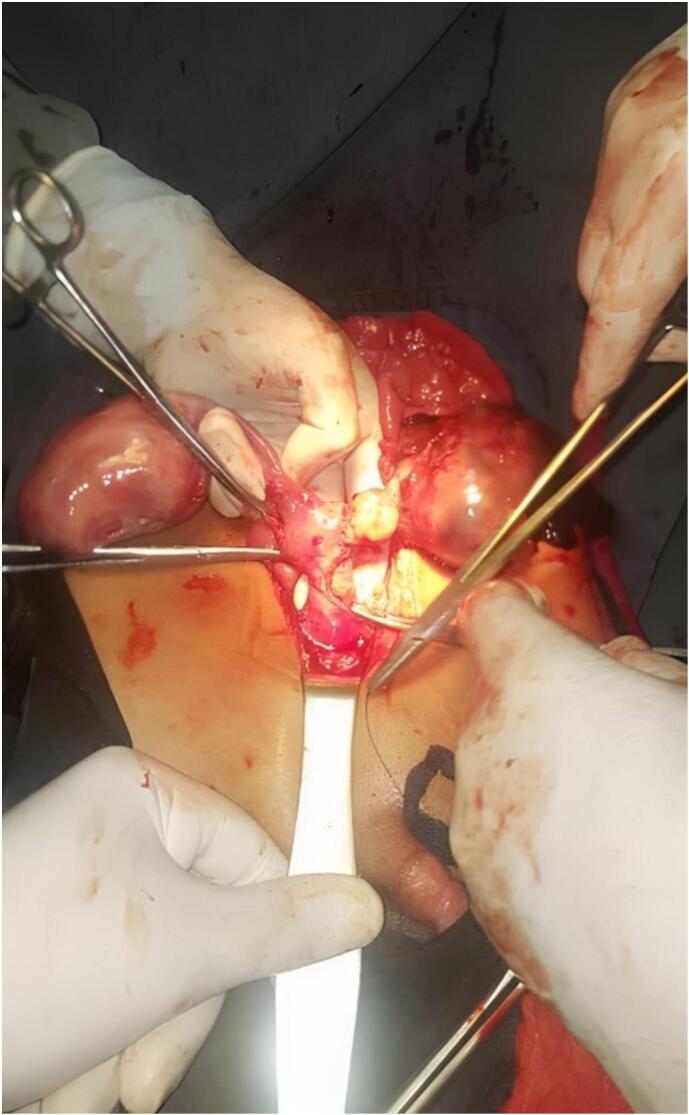
Intraoperative picture displays both ovaries with fibrotic characteristics.

**Fig. 2:**
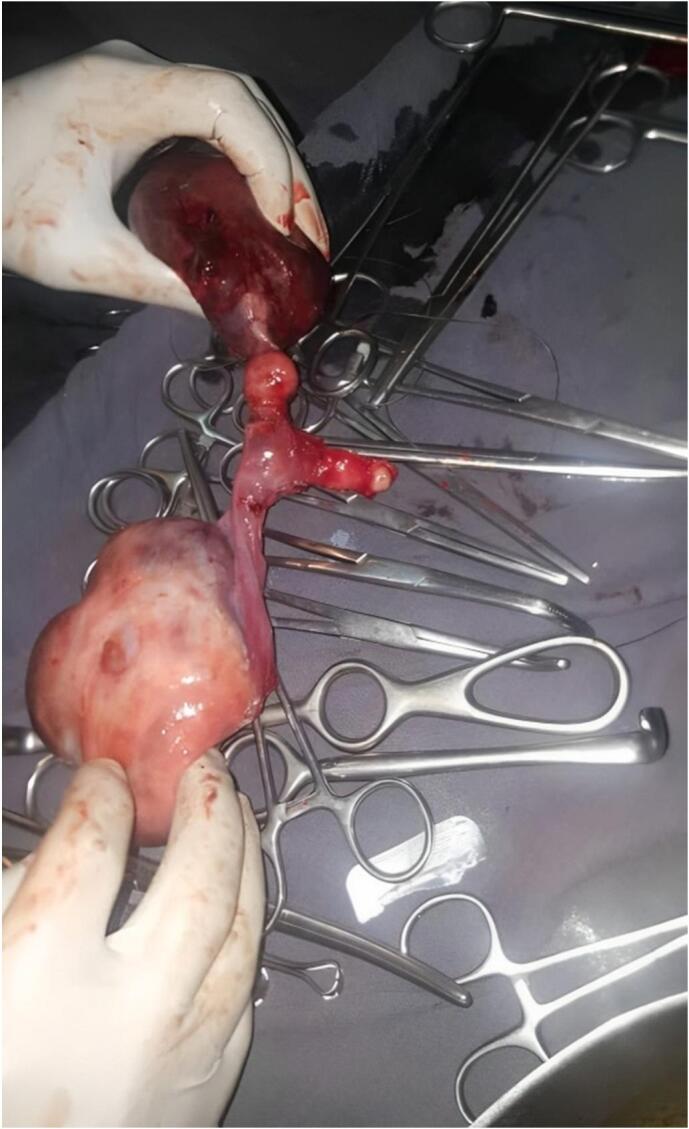
Postoperative image of surgically removed Müllerian duct structures.

A 14-year-old boy presented with an extensive medical history of abdominal pain, anorexia, weight loss, acute pain during the visit, and the presence of a palpable mass and swelling in the abdomen. The patient had previously received traditional herbal medicines and analgesics. He had a male phenotype and showed typical male external genitalia and secondary sexual characteristics. No obvious female genitalia or female secondary sexual characteristics were observed on physical examination. However, he had bilateral undescended testicles, and palpation of the abdomen revealed the presence of large masses on both sides of the abdominal cavity. Further examinations, including semen and hormonal analysis, computed tomography (CT), and ultrasound scans, were then performed. The scrotal ultrasound revealed the absence of both testicles. In semen analysis, he had azoospermia. The hormonal analysis conducted indicated a marked increase in LH levels and a low volume of testosterone, while the levels of FSH and AMH were found to be within the normal range. The abdominal ultrasound revealed normal-sized kidneys without any signs of hydronephrosis or the accumulation of free fluid in the peritoneal, pericardial, or pleural cavities. The urinary bladder exhibited a lumen devoid of echoes, with a wall thickness within normal limits, and devoid of lithiasis or mass lesions. Nevertheless, a multitude of intricate masses 7 cm in size, primarily cystic with septation and diffused low-level echoes, were visualized within the compressed visceral organs and bowel loops of the abdominal region. These findings were consistent with lymphangiomyoma. Additionally, an abdominopelvic CT scan revealed complex cysts with multiple cystic compartments and thick walls located in the posterior-superior region of the pelvic cavity, anterior to the rectum and sigmoid colon. A structure resembling testicles was additionally observed in the inguinal region. The patient received a diagnosis of bilateral undescended testicles and abdominal cysts, prompting referral to a urologist and an adolescent surgeon for further evaluation. After hospitalization in the general surgery department of the hospital, the patient was scheduled to undergo open cystectomy and, if possible, orchiopexy. However, during the surgical procedure, physicians discovered the presence of fallopian tubes, fibrotic like-ovaries, and a lump on the fallopian tube (Fig. 1), leading to the diagnosis of PMDS. Consequently, the planned cystectomy was abandoned, and a hysterectomy was performed instead. The MD was carefully removed (Fig. 2), and the testicles were carefully repositioned during an orchiopexy. The patient recovered normally, was closely monitored for two days, was discharged without complications, and was recommended for karyotyping and pathological examination. The fertility status of the patient was assessed during a follow-up examination conducted two months later. As an aspect of infertility, the analysis of sperm quality through spermography revealed poor semen quality. Consequently, he underwent a comprehensive evaluation carried out by an internist-urologist and received the necessary medications.

## 3 Discussion

Nilson's groundbreaking discovery in 1939 unveiled PMDS [1], a rare anomaly of sexual development manifested by the presence of Müllerian derivatives in individuals with a male phenotype and genotype [3]. During embryogenesis, the reproductive tract in both males and females consists of two ducts: the Müllerian duct and the Wolffian duct. In male fetuses at 8 weeks of gestation, Leydig cells and Sertoli cells in the newly formed testes produce testosterone and MIS, respectively. Testosterone plays a crucial role in the differentiation of the Wolffian duct into various structures, such as the epididymis, seminal vesicles, and vas deferens. Conversely, MIS, also known as AMH, is a glycoprotein homodimer responsible for testicular descent and regression of the Müllerian duct. In women, the lack of testosterone leads to degeneration of the Wolffian duct. Furthermore, the absence of MIS allows the Müllerian duct to differentiate into female reproductive organs, including the uterus, fallopian tubes, and ovaries [9]. PMDS exhibits two distinct anatomic variants, namely the male form and the female form. The male form is the more common variant, accounting for approximately 80–90 % of cases. It typically manifests as a hernia, uteri inguinal, or crossed testicular ectopia. Conversely, the female form is observed in only 10–20 % of cases and is characterized by bilateral undescended testicles [7]. The main cause of this disorder is the deficiency of AMH. This syndrome is caused either by a lack of production of AMH from Sertoli cells (PMDS type I) or by resistance to the AMH receptor (AMHR2 gene) (PMDS type II) [9]. The identification of PMDS is challenging due to its complicated and anatomically diverse features, as well as the lack of consensus on diagnostic criteria [1,6], and the absence of specific clinical symptoms, hormones, or ultrasound tests that could aid in identification. Furthermore, increasing the distinction between PMDS and partial testicular dysfunction increased the complexity of the diagnostic process. However, diagnosing PMDS in children is more challenging due to naturally elevated AMH levels in prepubescent boys, resulting in fewer cases being identified [2]. The identification of PMDS often occurs incidentally during surgical procedures for undescended testicles or inguinal hernias and occasionally during the management of infertility [3]. In our case, the patient had female-type PMDS.

The management of PMDS has been a topic of discussion. The primary considerations revolve around maintaining fertility and averting the development of malignancies. Three therapeutic theories have been suggested as potential approaches for the management of PMDS. The first theory proposed orchiopexy for UDT while preserving Müllerian structures. The second theory supports orchiopexy, the removal of Müllerian structures, and the correction of any associated anomalies. The proposed approach to eliminating all risks of malignancy by removing both the Müllerian structures and the cryptorchid testes is presented in the third theory [10]. We recommend the second course of treatment with orchiopexy to preserve fertility, eliminate the risk of Müllerian malignancy, and avoid lifelong follow-up with Müllerian excision. Additionally, it is important to consider the long-term growth, development, and fertility of patients with PMDS before removing nonfunctional MD structures [2].

## 4 Conclusion

PMDS is a condition that is often detected during medical examinations for problems such as undescended testicles and inguinal hernias. The condition is notable because Müllerian structures are susceptible to malignant transformation. Surgeons need to be aware of PMDS, as it can manifest during routine procedures. The proposed course of treatment includes performing a hysterectomy while preserving the vas deferens on both sides, which can help preserve fertility and minimize the risk of malignancy due to the persistence of Müllerian structures.

## Consent

Written informed consent was obtained from the patient's parents for publication and accompanying images. A copy of the written consent is available for inspection by the editor-in-chief of this journal upon request.

## Ethical approval

This case study received ethical approval from the authors' institute.

## Funding

None.

## Author contribution

Dawood Hossaini: Wrote Manuscript. Mohammad Mahdi Wahdat: preforme the surgery. Ali Ahklaqi: Collected Data. Murtaza Haidary: Revised Manuscript, led the study.

## Guarantor

Murtaza Haidary

Murtaza.daidary@knu.edu.af.

sadramurtaza@gmail.com.

0093(0)707927247.

0093(0)796473678.

## Declaration of competing interest

The authors disclose no conflicts of interest.
